# The Influence of Medical Expertise and Information Search Skills on Medical Information Searching: Comparative Analysis From a Free Data Set

**DOI:** 10.2196/62754

**Published:** 2025-04-17

**Authors:** Aline Chevalier, Cheyenne Dosso

**Affiliations:** 1 Laboratoire Cognition, Langues, Langage, Ergonomie (Centre National de la Recherche Scientifique UMR5263) Maison de la Recherche Université de Toulouse Toulouse France; 2 Laboratoire d’anthropologie et de psychologie cliniques, cognitives et sociales (UPR7278) Université Côte d'Azur Nice France

**Keywords:** information searching, credibility, internet, medicine, information search skills

## Abstract

**Background:**

Nowadays, the internet has become the primary source of information for physicians seeking answers to medical questions about their patients before consulting colleagues. However, many websites provide low-quality, unreliable information that lacks scientific validation. Therefore, physicians must develop strong information search skills to locate relevant, accurate, and evidence-based content. However, previous studies have shown that physicians often have poor search skills and struggle to find information on the web, which may have detrimental consequences for patient care.

**Objective:**

This study aims to determine how medical students and residents searched for medical information on the internet, the quality of the web resources they used (including their nature and credibility), and how they evaluated the reliability of these resources and the answers they provided. Given the importance of domain knowledge (in this case, medicine) and information search skills in the search process, we compared the search behaviors of medical students and residents with those of computer science students. While medical students and residents possess greater medical-related knowledge, computer science students have stronger information search skills.

**Methods:**

A total of 20 students participated in this study: 10 medical students and residents, and 10 computer science students. Data were extracted from a freely accessible data set in accordance with FAIR (Findable, Accessible, Interoperable, and Reusable) principles. All participants searched for medical information online to make a diagnosis, select a treatment, and enhance their knowledge of a medical condition—3 primary activities they commonly perform. We analyzed search performance metrics, including search time, the use of medical-related keywords, and the accuracy of the information found, as well as the nature and credibility of web resources used by medical students and residents compared with computer science students.

**Results:**

Medical students and residents provided more accurate answers than computer science students without requiring additional time. Their medical expertise also enabled them to better assess the reliability of resources and select high-quality web sources, primarily from hospital websites. However, it is noteworthy that they made limited use of evidence-based tools such as PubMed.

**Conclusions:**

Although medical students and residents generally outperformed computer science students, they did not frequently use evidence-based tools. As previously observed, they may avoid databases due to the risk of encountering too many irrelevant articles and difficulties in applying appropriate filters to locate relevant information. Nevertheless, clinical and practical evidence-based medicine plays a crucial role in updating physicians’ knowledge, improving patient care, and enhancing physician-patient relationships. Therefore, information search skills should be an integral part of medical education and continuing professional development for physicians.

## Introduction

### Internet as a Tool for Medical Information Searching by Physicians

Nowadays, searching for information on the web is an integral part of a physician’s professional activity. While colleagues were the primary source of information in 2006 [1], more recently, the internet has become the first source before consulting colleagues when physicians have medical questions about patients [2,3].

Efficient information searching has various positive outcomes for physicians and, by extension, their patients [2]. It may improve patient care [4,5], clinical decision-making [5], and physicians’ knowledge [5,6]. It also reduces delays in diagnosis, treatment initiation, and modification [5], as well as potential medical errors and unnecessary medical examinations [6].

More precisely, physicians, especially younger ones, use the internet to search for information for various purposes [1]: (1) finding information and solutions to specific patient diagnostic issues (reported by 33.7%-57% of respondents in the previous study [1]); (2) obtaining new information on diseases (20.4%); and (3) staying informed about the latest research on specific topics and general matters, such as diseases (27%), new therapies and treatments (8.8%), and drug dosing (5.6%), as also shown by [7-9].

Concerning information related to treatment and diagnosis, physicians take between 2 and 32 minutes to find answers on the internet, depending on the nature of the information sought (eg, side effects, drug dosing, new therapies) [2]. Nevertheless, most studies indicate that physicians spend less than 10 minutes searching for answers and less than 2 minutes during patient consultations [2]. During consultations, they primarily search for information on drug dosing and its potential side effects or to show images to patients [10].

### Difficulties in Finding Relevant Web Resources and Its Consequences

Although physicians reported finding information in most cases (>50% of searches for 70% of physicians) and over 60% were confident in their ability to find the information they needed on the internet [2], they still face various difficulties and barriers in information searching. For instance, a survey of general practitioners identified the main obstacles as a lack of information search knowledge or skills, clinical practice conditions (time pressure or patient-related concerns), information-related issues (information overload, quality concerns, or low relevance), and language barriers [11]. It is noteworthy that, occasionally, reliance on internet-supplied information can lead to clinical errors, as physicians may respond to and process such information—even when it contradicts their preexisting knowledge [12]—regardless of its accuracy. While the use of web resources can enhance physicians’ clinical decisions [6], occasional discrepancies in the quality and reliability of the information found may introduce errors in the decision-making process. Outpatient diagnostic error rates have been estimated at 5%, affecting more than 12 million individuals per year, while inpatient diagnostic errors range from 6% to 7%, with 20% of initial diagnoses being modified [13]. Efficient use of information access systems (eg, search engines) can positively impact patient care, but misuse of the internet may have the opposite effect. Therefore, physicians and medical students must develop accurate information search skills (ie, procedural knowledge of tools such as Google [Alphabet Inc] or PubMed [National Institutes of Health]) to use these systems effectively within the specificities of the medical domain.

Previous studies have reported a lack of information search skills, requiring technical support for information retrieval [1], unawareness of accessible sources [2], difficulties in accessing reliable and up-to-date medical information, and challenges in developing efficient search strategies [4]. Additionally, on the internet, physicians face the challenge of processing large amounts of information in a very short time [14].

Previous studies have shown that well-designed, easy-to-navigate websites are generally considered more reliable than those with a confusing or complex interface [15]. The first websites listed by search engine tools are often perceived as more credible than those appearing later [16]. However, neither a well-designed website nor a high search engine ranking guarantees that the information provided is credible and reliable. Although physicians require reliable and relevant documents to integrate into clinical practice, the internet presents a vast array of medical-related information, not all of which is verified. For instance, a study found that only 58% of eHealth websites in the United States met the criteria for content accuracy and credibility [17]. Consequently, physicians must develop information search skills to effectively find and assess web resources.

However, an institutionalized perception persists that the information found does not significantly enhance medical practice [6]—for a review, see [2]. This perception may be partly because not all physicians are aware of the availability of evidence-based medical resources on the internet (eg, PubMed) or they may lack the search skills to access them [18].

### Main Web Resources Used

To effectively improve physicians’ information search skills, it is essential to understand how medical students search for medical information and which web resources they use during their university program. A study on medical students found that they searched for online medical and clinical information daily (2-3 times per day) [19]. They accessed numerous websites (>50), including some recommended by the NHS (National Health Service), such as PubMed, which was used by only 30% of physicians and accounted for just 8% of all their searches. However, they also relied on many general websites, particularly Wikipedia (Wikimedia Foundation; used by 71% of students and accounting for 26% of searches), patient forums, medical-specific wikis, and Google (used by 79% of students and comprising 32% of all searches). Indeed, Google is the most widely used general web search engine and has become an important tool in physicians’ searches. Nevertheless, it is not specialized in medicine, and numerous websites present medical-related information without being reviewed by scientists or physicians [20,21], often leading to low-quality information [22]. Consequently, physicians must carefully evaluate the quality of information obtained from websites. Furthermore, physicians and medical students often prefer using Google over medicine-specific databases to answer clinical questions [23]. For instance, Krause et al [23] provided clinical questions to emergency department residents and asked them to search for answers either using a search engine (Google) or without it (by searching in a real environment). The results showed that while the number of incorrect answers increased with Google, the number of unsure responses decreased compared with searches conducted without Google. The residents developed a false sense of confidence in their answers and demonstrated poor efficiency in searching for and identifying correct clinical information using Google. While students and physicians may perceive Google as an evidence-based medical tool, it is not inherently an evidence-based medical database like PubMed.

Based on these prior studies, we can assert that medical-related information available on the internet can support clinical decision-making in patient health care, provided that physicians and medical students are trained to evaluate the quality of sources and content. The quality of medical-related information can be assessed according to 2 dimensions: (1) The credibility of sources, which refers to the degree of trustworthiness associated with the information and its origin [24-27]. This includes evaluations of accuracy, authority, objectivity, currency, and coverage [26,28]. Credibility is closely related to trustworthiness, reliability, accuracy, authority, and quality [24,25,29], although not always inseparable (eg, reliable information can sometimes be found on sites with low credibility). Consequently, credibility may influence the perceived reliability of information. (2) Reliability, in turn, refers to the extent to which information remains consistent and trustworthy under similar circumstances [30,31].

In the medical domain, Mikalef et al [6] classified credible documents as follows: (1) Authoritative web documents include scientific digital databases (eg, PubMed), websites of medical organizations (eg, the European Society of Cardiology), online peer-reviewed scientific journals (eg, The Lancet, JAMA), specialized medical journals (eg, *Circulation*, *Heart*), e-books, and government agency websites (eg, the Public Health Administration, the Food and Drug Administration). (2) Nonauthoritative web documents include medical portals, personal websites of physicians, pharmaceutical company websites, social media, and medical equipment websites. While authoritative sources can generally be considered credible and reliable, nonauthoritative sources require in-depth evaluation to determine their credibility and reliability. Therefore, when physicians and medical students use general websites to find medical-related information, they must:

- Translate a clinical or medical question into a search strategy to locate reliable information. This requires using specific medical terms or selecting credible web resources (eg, scientific databases).
- Evaluate credibility, reliability, relevance, and accessibility to determine which web resources to consult and use (see the review by [2]).

These 2 activities can be highly complex, especially when using general-purpose search engines (eg, Google), as individuals must select appropriate keywords and identify credible websites to obtain reliable information relevant to their professional objectives.

To this end, medical students and residents can rely on their prior domain knowledge to navigate the web more effectively. However, they often lack information search skills and familiarity with scientific tools and search systems, which are critical for efficient information retrieval. Users with strong information search skills are better guided by the components of the search space, regardless of the domain in which they seek information [32], a phenomenon known as the cross-domain transfer of information search skills [33]. Specifically, such users demonstrate more adaptive search behaviors tailored to their tasks and employ advanced search strategies [34]. For instance, they are more likely to incorporate Boolean operators into their queries to construct complex search equations rather than relying solely on simple keyword searches [35,36]. Additionally, they effectively utilize indexes and thesauri provided by search systems to identify relevant vocabulary, allowing them to refine their queries with greater specificity and precision as their search sessions progress [32].

However, not all academic programs place equal emphasis on developing information search skills. For example, computer science students acquire more advanced operational search skills compared with students in other fields [36,37]. Consequently, computer scientists have a deeper understanding of search systems and use them more effectively—leveraging features such as recommendations, thesauri, and domain-specific scientific databases—to conduct advanced searches, expand their vocabulary, and achieve their research objectives [38]. These search skills enable them to construct more structured and precise queries as their searches evolve [39].

In summary, prior domain knowledge facilitates attentional guidance as well as the reading and comprehension of web-based information [40]. By contrast, prior information search skills enhance the effective use and understanding of information systems.

### Research Objectives and Questions

This study aimed to examine the effects of domain-specific prior knowledge versus information search skills on information search behaviors and performance. While medical students and residents benefit from their medical expertise when conducting medical searches, they lack the information search skills of their computer science counterparts, and vice versa. Identifying performance indicators and the challenges faced by these distinct user profiles will help clarify the limitations of relying solely on domain knowledge in medical information searches.

More precisely, most prior studies examining how physicians search for information have relied on interviews or questionnaires to assess their search behaviors [2]. However, very few have analyzed the actual search activities of physicians or medical students and residents, despite the importance of understanding how they navigate the web when conducting necessary searches. To address this gap, we conducted an experimental study in which medical students and residents were compared with computer science students in their web-based information searches. More specifically, this study aimed to determine whether prior medical knowledge enabled medical students and residents to achieve higher search performance and make better choices regarding reliable and credible resources compared with computer science students, who had greater experience in information search and higher search skills.

More specifically, this study aimed to provide insights into the following research questions (RQs):

- RQ1: Do the most common searches conducted by physicians—treatment, diagnosis, and learning about a specific medical topic—result in different search performances between computer science students (who have higher information search skills but less medical knowledge) and medical students and residents (who have greater medical knowledge but lower information search skills)?
- RQ2: What types of resources do they consult during medical search tasks, and how do they assess the credibility of these resources based on their domain expertise (medicine vs information search)?
- RQ3: Do these 2 groups of students evaluate the reliability of the resources they use and the quality of the answers they provide differently when performing a medical search task?

## Methods

### Recruitment

The freely accessible data set used in this study was extracted from a user study conducted by Dosso et al [41] during the COVID-19 lockdown. The data set is available online [42].

A total of 20 students from the University of Toulouse participated in this study:

- 10 medical students and residents in medicine: 5 females and 5 males (mean age 25.4 years, SD 3.1 years); 2 were pursuing a master’s degree and 8 were in an MD (Doctor of Medicine) program.
- 10 computer science students: 4 females and 6 males (mean age 23.8 years, SD 1.5 years); 5 were pursuing a master’s degree and 5 were PhD (Doctor of Philosophy) students.

To evaluate their level of knowledge in medicine and their familiarity with the internet, all the participants had:

- To self-evaluate their knowledge in medicine, on a 5-point Likert scale ranging from 1 (very low) to 5 (very high). Medical students self-evaluated their level of medical knowledge higher than computer science students (mean_med_ 4.2, SD 0.63; mean_cs_ 1.3, SD 0.48; *t*_18_=–11.5, *P*<.001, *d*=–5.15).
- To complete a multiple-choice questionnaire in the Medicine domain, elaborated by a physician, which consisted of 10 questions, each with 5 response options (1 correct, 3 incorrect, and 1 “I don’t know”). Only correct answers earned 1 point, so the score per participant could range from 0 to 10. Results indicated that student-residents in medicine scored significantly higher (mean 5, SD 0.66) than students in computer science (mean 0.5, SD 0.97; *t*_18_=–12.1, *P*<.001, *d*=–5.4).
- To complete the Information Search Self-Efficacy Scale developed by Rodon et al [43] and indicate the number of years of internet use and the contexts in which it is used. Results showed that students in computer science (mean 4.4, SD 0.51) had higher information search self-efficacy scores than those in medicine (mean 3.1, SD 0.73; *t*_18_=4.56, *P*<.001, *d*=2.04). However, there was no significant difference in the number of years of internet use between the 2 groups (mean_CS_ 12.3, SD_CS_ 1.83 and mean_med_ 14, SD_med_ 2.79; *t*_18_=–1.61, *P*=.12, *d*=–0.721). All the participants reported using the internet for both university and personal purposes.

### Ethical Considerations

Before starting the search tasks, all participants received a detailed explanation of the study’s objectives and procedures. Informed consent was obtained from each participant through a written agreement, which outlined the study’s purpose, potential risks and benefits, data usage protocols, and participant rights, including the right to withdraw at any time without consequence. This study was reviewed and approved by the Ethics and Research Committee of the University of Toulouse (approval/project number 2021-354).

### Procedure and Material

First, all participants completed an online prequestionnaire that included a free and informed consent form, questions on sociodemographic variables (eg, age, gender, education level, native language), internet information search scales, and multiple-choice questions assessing their level of medical knowledge. Additionally, participants described their general internet habits (ie, browsing device used, preferred browser, and search engine) and completed the Information Search Self-Efficacy Scale [43].

Then, the participants conducted the experiment at home during the COVID-19 lockdown in 2020-21. The experimental materials included the following:

- A printed booklet with general instructions for the experiment, a training exercise on using the logging browser, statements for the search tasks, and designated spaces for writing answers. As the written production task was conducted with pen and paper, the computer was used only for the search sessions.
- A memory stick containing the browser was provided for the search sessions, enabling participants to directly search for information while reviewing the experimental material.

Participants were instructed to complete each search task independently, without taking notes during the search session. This ensured that their final answers primarily reflected the information retained in memory after the search.

Both groups of participants (medical students/residents and computer science students) were presented with 3 distinct search tasks in general medicine (see Table 1 for detailed task descriptions). These tasks reflected real-world scenarios commonly encountered in medical practice, such as (1) enhancing knowledge in a specific disease area, (2) searching for information related to a particular patient problem, and (3) identifying and selecting the most appropriate treatment options for patients.

These scenarios are well-documented in medical research, as supported by previous studies [1,2].

The instructions specified that participants had to write their answers for each task only after completing the search session (ie, after closing the browser). There was no time limit. Note-taking was not permitted during the search session to ensure that responses closely reflected the knowledge retained in participants’ memory.

Three different search tasks in general medicine were assigned to the participants (medical students/residents and computer science students; see Table 1 for the search tasks). These tasks closely resembled real-world scenarios encountered by physicians and medical students, such as improving their knowledge in a specific disease area, searching for information on a particular patient problem, and determining the most appropriate treatment for patients [1,2].

**Table 1:** The 3 different tasks provided to the participants and correct answers.

Three different tasks and answers			Tasks’ instructions		Subtasks required
Learning task					
	Purpose: Improving knowledge of a specific area/disease Correct answer: No correct answer was defined; instead, participants’ responses were analyzed to compute the number of relevant elements they provided.	You want to learn more about endometriosis.		Understanding endometriosis Understand the issues related to endometriosis Discover/learn more about medical solutions to treat endometriosis	
**Treatment decision task**					
	Purpose: Choosing the most appropriate treatment Correct answer: Anticoagulant treatment (because the old woman did not fall too many times for her age)	An 83-year-old woman had a nonsequelae stroke 5 months ago. At the stroke assessment, atrial fibrillation was discovered. She had fallen 3 times in the last 2 months. Should anticoagulant therapy be initiated? After evaluating the benefit-risk ratio of initiating or not initiating anticoagulant treatment, select the management option that you find most appropriate and justify your choice.		Understanding nonsequential stroke Understanding atrial fibrillation Understanding the risk of falls Identify the advantages and disadvantages of anticoagulant therapy Judge this information according to the criteria to be established (benefit/risk assessment)	
**Diagnosis task**					
	Purpose: Determining the pathology Correct answer: Splenic infarction or spleen infarction	A 47-year-old man presented to the emergency department with left hypochondrium pain that had been evolving for 24 hours and was not relieved by level 1 analgesics. His history included cutaneous lupus and polycythemia. The biological workup was normal. The abdominopelvic scanner found 2 splenic hypodensities. With the information collected on the internet, propose your diagnosis and etiological hypotheses		Understanding left hypochondrium Understanding of level 1 analgesics Understanding cutaneous lupus and polycythemia Understanding splenic hypodensity Analyze the information previously collected and propose a diagnosis with etiological hypotheses	

### Variables

Two independent variables (IVs) were manipulated:

- IV1: Domain expertise of participants (medical students/residents vs computer science students) as a between-participants variable.
- IV2: Search tasks (learning, choosing treatment, diagnosis) as a within-participants variable.

A total of 6 dependent variables (DVs) were measured to bring some answers to the 3 RQs. For RQ1, we measured 3 DVs:

- DV1: Total time (seconds) spent on each search task, measured from the first query entered to the completion of the multiquery process.
- DV2: Number of medical-related keywords generated by participants, excluding those explicitly provided in the search task instructions.
- DV3: Accuracy and relevance of retrieved information:
  - For diagnostic and treatment tasks, responses were scored as 0 (incorrect or abandoned) or 1 (correct).
  - For the learning task, responses were scored from 0 to 4 based on the amount of relevant information provided and interconnected. Relevant information was defined as content directly related to the assigned topic (endometriosis) in accordance with the information context [44]. This is rated as follows: 0 (incorrect information), 0.5 (correct information), 1 (correct and relevant information), 1.5 (correct and relevant information linked to general vocabulary), 2 (correct and relevant information linked to medical vocabulary), 3 (correct and relevant information linked to general vocabulary and interconnected), and 4 (correct and relevant information linked to medical vocabulary and interconnected).

For RQ2, we measured:

- DV4: Evaluation of the credibility and type of web resources consulted:
  - DV4a: Credibility score of the websites visited for each task.
  - DV4b: Distribution of the types of web resources consulted, categorized as websites or as documents.

For DV4a, the credibility of web resources consulted per search task was scored as follows, based on Mikalef et al [6]:

- Peer-reviewed scientific journals or scientific databases (eg, PubMed, ScienceDirect, The Lancet)=2 points per URL
- Documents from hospitals, health authorities (eg, World Health Organization), and government agencies=1.5 points per URL
- Health-related websites verified by HONcode (Health On the Net code; certification by the nonprofit Health On the Net Foundation [45])=1 point per URL
- Other websites (not scientifically verified)=0 points per URL

For DV4b, we analyzed the distribution of resources consulted across different categories (peer-reviewed scientific journals, hospital/government documents, HONcode-certified websites, and other websites) during each search task. This distribution was based on the number of resources consulted per category (as defined in DV4a) and per search task.

For RQ3, 2 DVs were measured:

- DV5: Self-assessment of reliability of web documents visited while achieving the search task [46]: “Overall, the search engine results, websites, or documents (results from Google, websites, pdf, etc) consulted were: (1) absolutely not trustworthy, (2) very untrustworthy, (3) not very trustworthy, (4) moderately trustworthy, (5) somewhat trustworthy, (6) trustworthy, or (7) extremely trustworthy. The higher the score was, the more participants perceived web documents as reliable.”
- DV6: Self-assessment of the quality of their own answers on a 4-point scale for the 3 tasks. Participants rated their perceived answer quality as follows: (1) very bad, (2) bad, (3) good, and (4) very good. A higher score indicated a more favorable self-evaluation of their answers.

### Statistical Analysis

For all DVs, we compared student-residents in medicine with students in computer science for each task using either parametric or nonparametric tests. Specifically, for all DVs except DV4b, we performed Student *t* tests. For DV4b, we conducted a repeated-measures ANOVA. Tests for homogeneity of variance (Levene test) and normality (Shapiro-Wilk test) were performed before conducting ANOVA and unpaired (1-tailed) *t* tests. Partial η^2^ was used as an index of effect size. When homogeneity of variance was violated, we applied the Mann-Whitney test. Additionally, some quantitative results are illustrated with qualitative examples.

## Results

### Overview

The results are presented according to the 3 RQs.

- *RQ1: Do the most popular searches developed by physicians (ie, treatment, diagnosis, and learning on a specific medical topic) lead to different search performances between computer science students (with higher information search skills but lower medical knowledge) and medical student-residents (with higher medical knowledge but lower information search skills)?*

Table 2 presents the dependent variables for RQ1.

**Table 2:** Dependent variables for RQ1 with regard to the search task and expertise domain.

Group of students			Learning task, mean (SD)		Diagnostic task, mean (SD)		Treatment task, mean (SD)
**DV1: Search time (seconds)**							
	Medicine	548 (474)		705 (444)		634 (407)	
	Computer science	520 (373)		1099 (1085)		936 (699)	
**DV2: New keywords related to medicine (mean number)**							
	Medicine	1.80 (0.91)^a^		4 (4.19)		4.8 (4.13)	
	Computer science	0.3 (0.48)^a^		2 (2.35)		2.7 (3.97)	
**DV3: Correct answers or relevant information provided (mean number)**							
	Medicine	3.9 (0.31)^a^		0.9 (0.31)^a^		0.85 (0.15)^a^	
	Computer science	3.5 (0.52)^a^		0 (0)^a^		0.1 (0.31)^a^	

^a^Significant differences between medical students/residents and computer science students.

### Time Spent to Find Information

Regarding the time required to find information, no significant differences were observed between student-residents in medicine and those in computer science for any task (all *t* tests were nonsignificant: *t*_18_=-.146, *P*=.88 for the learning task; *t*_18_=1.06, *P*=.30 for the diagnostic task; and *t*_18_=1.18, *P*=.25 for the treatment task).

### Keywords Related to Medicine Added by the Participants

Student-residents in medicine added more medicine-related keywords than students in computer science, but only for the learning task (*t*_18_=–4.57, *P*<.001, *d*=2.04). On average, student-residents in medicine added 1.6 keywords (SD 0.91), compared with 0.3 (SD 0.48) for students in computer science. The keywords added by student-residents in medicine were primarily related to specific websites they were familiar with (eg, associations of doctors in gynecology or government health agency websites, where they could find medical information).

For the treatment and diagnostic tasks, statistical analyses revealed no significant differences between student-residents in medicine and students in computer science (*t*_18_=–1.32, *P*=.20, and *t*_18_=–1.16, *P*=.26, respectively).

Correct information retrieval for the diagnostic and treatment tasks, as well as the number of relevant information elements provided by participants for the learning task, was analyzed. The results are as follows:

For the treatment and diagnostic tasks, medical student-residents provided more correct answers than those in computer science.

- For the treatment task (*t*_18_=–6.77, *P*<.001, *d*=–3.03), the mean score was 0.85 (SD 0.15) for medical student-residents, compared with 0.1 (SD 0.31) for computer science students.
- For the diagnostic task (*t*_18_=–9, *P*<.001, *d*=–4.02), the mean score was 0.9 (SD 0.31) for medical student-residents, compared with (SD 0) for computer science students.

For the learning task, even after applying the square root correction (√^2^), the homogeneity of variance was violated. Therefore, we applied the nonparametric Mann-Whitney test. Medical student-residents provided more relevant information elements than computer science students (*U*_18_=30, *P*=.03, rank biserial correlation=0.4), with a mean of 3.9 (SD 0.31) for medical students and 3.5 (SD 0.52) for computer science students.

As illustrated in Multimedia Appendix 1 with 4 examples, the nature of the information elements provided for the learning task varied depending on the participants’ domain of expertise. This task was exploratory and was approached differently based on the participants’ expertise. Medical student-residents generally provided comprehensive responses specific to medicine, including definition and prevalence, symptoms, causes and consequences, diagnosis, and possible treatments. By contrast, computer science students primarily focused on definition, prevalence, and symptoms, with very few mentioning possible treatments. Their answers were more superficial compared with those of medical student-residents, who applied a medical framework to obtain information about the pathology.

- *RQ2: What types of resources do participants consult, and what is their credibility level when performing medical search tasks, considering their domain of expertise (medicine vs information search)?*

### Credibility Resources Consulted, Scores, and Websites Used

First, regarding the credibility scores obtained (DV4a), medical student-residents scored higher than computer science students for the learning task, with a mean of 5.85 (SD 4.37) compared with 2.2 (SD 2; *t*_18_=–2.40, *P*=.046, Cohen *d*=1.08). For the diagnostic task, only a marginal effect was observed (*t*_18_=–1.48, *P*=.07), with a mean of 13.45 (SD 9.63) for medical student-residents versus 8 (SD 6.53) for computer science students.

For the treatment task, no significant difference was found between the 2 groups, with a mean of 6.9 (SD 4.67) for medical student-residents and 5.4 (SD 4.09) for computer science students (*t*_18_=–0.76, *P*=.45).

**Figure 1:**
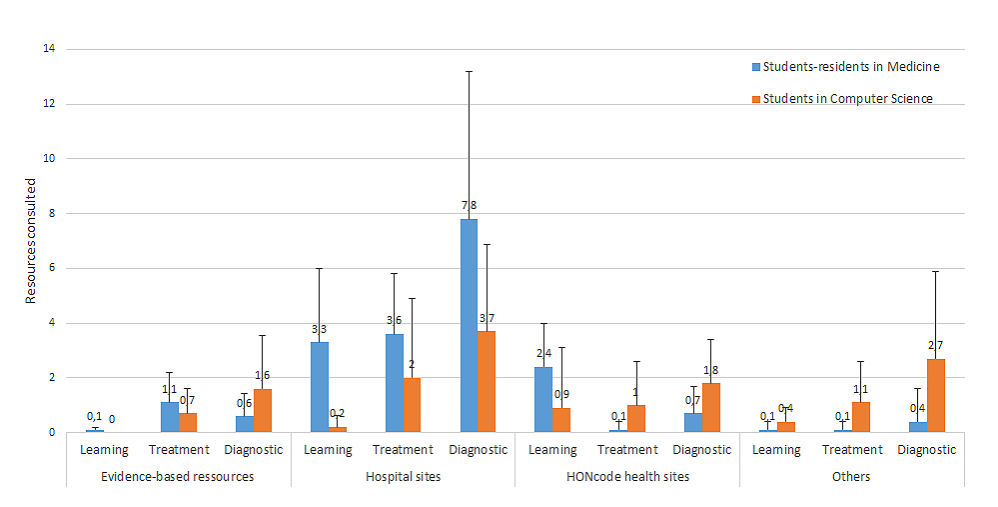
Means (and SDs) of web resources consulted with regard to the expertise domain of students.

Next, we analyzed the distribution of internet resources used (DV4b; Figure 1). The resources did not hold the same prominence during the information search (*F*_3,54_=28.12, *P*<.001, η^2^p=0.61); hospital websites (*P*<.001) and documents (*P*<.001) were used more frequently than other sources.

We conducted a more detailed analysis of resource usage based on expertise and tasks. We observed:

- For evidence-based resources, surprisingly, no significant difference was found between medical student-residents and computer science students (*F*_1,18_=0.31, *P*=.58). These resources were rarely visited by any participants.
- For hospital websites, medical student-residents used them more frequently than computer science students (*F*_1,18_=13.96, *P*<.001, η^2^p=0.44).
- For HONcode health websites, computer science students consulted these resources more frequently than medical student-residents (*F*_1,18_=6, *P*=.02, η^2^p=0.25).
- For other websites, computer science students used them more frequently than medical student-residents (*F*_1,18_=5.21, *P*=.001, η^2^p=0.34).

Next, qualitative analyses examined the main websites accessed through opened URLs (see Table 3).

**Table 3:** The main websites visited by participants and their score with regard to the search task to be performed and expertise.

Tasks and websites (resources)				Authority versus nonauthority websites and scores obtained
**Learning task**				
	Student-residents in medicine	A government health agency [47] Association of Physicians in Gynecology [48]	Authority; score=1.5 points Authority; score=1.5 points	
	Students in computer science	Association of Patients With Endometriosis [49] Nonofficial health websites (eg, [50,51])	Nonauthority; score=1 points (verified by HONcode) Nonauthority; score=0 points	
**Diagnostic task**				
	Student-residents in medicine	Digital medical campus (not updated since 2016) [52] A university hospital [53]	Authority; score=1.5 points Authority; score=1.5 points	
	Students in computer science	[54-56]	Nonauthority; score=1 point (verified by HONcode) For [55,56]: nonauthority; score=0 points	
**Treatment task**				
	Student-residents in medicine	A Swiss medical journal [57] A government health agency [47]	Authority; score=2 points Authority; score=1.5 points	
	Students in computer science	A Swiss medical journal [57] The other websites consulted are very different from one participant to another	Authority; score=2 points

- *RQ3: Do they assess differently the reliability of resources they choose to use and the quality of answers they provided for the search task to be performed?*

Results of the self-assessment of the reliability of web documents consulted when answering this question at the end of each session (maximum of 7 points): a significant difference was found between medical student-residents and computer science students for the learning task (*t*_18_=–3.13, *P*=.003, *d*=–1.39), with a mean of 6.8 (SD 0.42) for medical student-residents and 5.8 (SD 0.91) for computer science students. For the treatment and diagnostic tasks, statistical analyses did not reveal any significant differences between the 2 groups (*t*_18_=–1.24, *P*=.12, and *t*_18_=–1, *P*=.16; Table 4).

**Table 4:** Dependent variables for research question 3 with regard to the search task and expertise domain.

Research question 3 dependent variables and group of students			Learning task, mean (SD)		Diagnostic task, mean (SD)		Treatment task, mean (SD)
**DV5: Self-assessment of resource reliability consulted (maximum score=7 points)**							
	Medicine	6.8 (0.42)^a^		6 (0.66)		5.4 (0.51)	
	Computer Science	5.8 (0.91)^a^		5.6 (1.07)		5.1 (0.56)	
**DV6: Self-assessment of quality of answer (maximum score=4 points)**							
	Medicine	1.84 (0.14)^a^		1.73 (0.13)^a^		1.79 (0.11)^a^	
	Computer Science	1.76 (0.08)^a^		1.31 (0.29)^a^		1.70 (0.10)^a^	

^a^Significant differences between medical students/residents and computer science students.

Results of the self-assessment of the quality of answers provided on a 4-point scale for the 3 tasks; participants rated their perceived answer quality on a scale from 1 (very bad) to 4 (very good).

For all search tasks, medical student-residents rated the quality of their answers higher than computer science students:

- For the learning task, medical student-residents had a mean self-assessed answer quality of 3.5 (SD 0.52), compared with 3.1 (SD 0.31) for computer science students (*t*_18_=–2.05, *P*=.03, *d*=–0.92).
- For the diagnostic task, medical student-residents had a mean self-assessed answer quality of 3.2 (SD 0.42), compared with 2.9 (SD 0.31) for computer science students (*t*_18_=–1.8, *P*=.04, *d*=–0.80).
- For the treatment task, medical student-residents had a mean self-assessed answer quality of 2.9 (SD 0.31), compared with 1.8 (SD 0.78) for computer science students (*t*_18_=–4.09, *P*<.001, *d*=–1.83).

## Discussion

### Principal Findings

Prior studies have suggested that physicians’ primary challenge when searching for information online is their lack of information search skills [2,23], which may negatively impact the quality of information found and, consequently, patient care. This study aimed to examine the strategies used by medical students and residents when searching for medical information online, the web resources they consulted (including the nature and credibility of these resources), how they assessed the reliability of these resources, and the quality of the answers they provided. The search behaviors of medical students and residents were compared with those of computer science students. While medical students and residents had greater medical knowledge, computer science students exhibited stronger information search skills but lower medical-related knowledge. The main findings indicated that medical students and residents outperformed computer science students. Overall, they found more relevant information, used more credible resources, rated these resources as more reliable, and had greater confidence in their answers than computer science students. Two particularly surprising and interesting results emerged: (1) medical students and residents generated more medical keywords than computer science students. These keywords, at least in part, helped them locate familiar websites, making them indirectly relevant to information searching; and (2) medical students and residents rarely used evidence-based resources, instead relying on familiar medical websites, such as hospital websites.

### Interpretation of the Results Obtained

First, regarding information search performance, the results confirmed expectations: medical students and residents outperformed computer science students. They provided more correct answers for treatment and diagnostic tasks and included more relevant and structured elements in learning tasks—all without requiring additional time. Their deeper understanding of the search topics enabled them to deliver more structured and comprehensive responses. For example, medical students and residents frequently included details on symptoms, causes, diagnosis, and possible treatments. By contrast, computer science students’ responses tended to be more superficial, focusing primarily on general statistics and potential consequences. These findings align with prior research on the positive effects of domain knowledge in generating more accurate and higher-quality search outcomes, regardless of the field of expertise [58-60]. Indeed, when processing information, individuals with domain expertise engage in top-down processing, which enhances reading comprehension and supports a deeper understanding of the content [61-63]. As a result, at the search outcome stage, domain experts can more effectively reuse extracted information to achieve their goals and produce higher-quality responses [60]. The advantages of domain expertise in medical information searches were also evident in keyword selection. Notably, medical students and residents used more medical-related keywords, particularly when researching a specific disease. These findings align with previous studies showing that medical experts employ a more specialized medical vocabulary than nonexperts [63-65]. The medical knowledge applied during query formulation allowed physicians to locate more precise information at a search speed comparable to that of computer science students. However, despite their speed, computer science students’ searches tended to be far less precise.

Regarding these initial findings—the quality of final responses, keyword formulation, and search precision—it is worth noting that medical students accurately self-assessed their responses. Their higher confidence in the reliability of their answers, compared with computer science students, likely stemmed from their reliance on familiar medical websites. This underscores their ability to effectively locate and utilize credible information from online and hospital-based resources, as previously demonstrated [2].

When comparing the objective credibility scores of consulted web resources with participants’ self-assessed reliability of those resources, the results showed that medical students and residents accessed more credible websites than computer science students for learning and diagnostic tasks. A significant difference was observed for learning tasks, while scores were comparable for diagnostic tasks. Learning and diagnostic tasks may require greater medical expertise to identify credible and reliable sources compared with treatment-related tasks. Accurate diagnosis often involves a careful differential diagnosis, requiring the exclusion of other conditions with similar symptoms. This process requires access to credible medical resources, as general websites are often insufficient. Medical students and residents effectively relied on reputable medical websites to access relevant case studies and other pertinent information. Likewise, learning about specific topics required current and reliable information, underscoring the need for high-quality medical sources.

A detailed analysis of the consulted websites revealed task-dependent differences between medical students/residents and computer science students. Notably, evidence-based resources were rarely used by any participants and were primarily consulted for diagnostic and treatment tasks. This finding was unexpected, as medical students and residents, given their rigorous training, were expected to prioritize evidence-based resources. While this result aligns with previous research highlighting the limited use of evidence-based resources among medical practitioners [66,67], it raises concerns about the effectiveness of current training programs in fostering evidence-based practice skills. As previously noted, physicians often struggle to formulate effective search queries in databases such as PubMed, leading to the retrieval of numerous irrelevant articles [68]. When using general search engines, they face 2 main challenges [11]: (1) managing and processing a vast amount of information and (2) assessing the reliability of content based on scientific evidence, all within a limited time frame. On average, physicians spend between 9 and 11 minutes searching the internet for medical information [2], a trend also observed among the medical students and residents in this study. To navigate these challenges, physicians tend to rely on familiar websites rather than exploring new content or consulting databases like PubMed [1,6,19].

The preference for familiar hospital websites observed in our study may offer a more streamlined and efficient—though potentially less comprehensive—pathway to relevant information. Our findings further support this trend, as medical students and residents consistently relied on familiar hospital resources across all search tasks, favoring established sources over broader exploration. In our experimental study, medical students and residents primarily consulted hospital-based resources for all tasks and did so more frequently than computer science students. This observation aligns with previous research showing that health care professionals frequently rely on a limited set of preferred online resources [1,6,19]. One study found that physicians visit familiar websites to reduce search time [18], often going directly to trusted medical sources [69-71]. They apply various criteria to assess information quality (usefulness and accuracy) and authority (trustworthiness and credibility) [19], which may be particularly relevant when evaluating hospital websites and documents. Additionally, physicians tend to search for methodical and empirically grounded medical solutions to minimize errors in decision-making while maximizing diagnostic and therapeutic effectiveness, as previously demonstrated [6]. By contrast, our study found that computer science students relied more on HONcode-certified health websites and other unverified sources. Despite their advanced search skills, they may struggle to assess the credibility of medical websites or fully comprehend the information presented on platforms such as PubMed and hospital websites. Their limited medical knowledge likely hindered their ability to identify relevant and accurate medical information.

Consequently, the medical expertise of students and residents allowed them to achieve higher scores, evaluate resources more effectively, and select more relevant web sources compared with computer science students.

### Implications

The results we obtained may have several implications.

First, a surprising finding was the limited use of evidence-based resources such as PubMed by medical students and residents. This may be due to the challenge of filtering relevant information from a vast number of irrelevant results, as previously documented [68]. Additionally, difficulties in effectively using search filters within these databases may have further hindered their ability to retrieve pertinent information. This finding underscores the need for enhanced training programs to equip health care professionals with the skills necessary to effectively utilize evidence-based resources. Integrating clinical and practical evidence-based medicine principles is crucial for updating medical knowledge, improving patient care, and fostering strong physician-patient relationships [72]. A study by the Institute for Healthcare Informatics [73] highlighted that physicians frequently rely on Wikipedia as a primary source of health care information, further emphasizing the need for targeted training initiatives. These programs should focus on strengthening information literacy skills, including the effective use of evidence-based databases such as PubMed and the critical evaluation of online health information. This could include instruction on HONcode principles, which help distinguish reliable from unreliable sources based on factors such as authorship, funding sources, and URL domains (.org, .gov, .net). Beyond their clinical practice, physicians who are well-versed in evaluating online information can also better support their patients. Many patients seek medical information on the internet, often without the ability to assess its accuracy [74], which can have negative consequences for their health care. Equipping physicians with the skills to identify credible and effective online resources would enable them to guide patients in interpreting the information they find online, ultimately improving patient education and decision-making. Physicians at all stages of their careers must stay updated on advancements in research, treatments, and best practices to ensure safe and effective patient care. Online scientific databases are essential tools in this process, particularly as the use of generative artificial intelligence (AI), such as ChatGPT (OpenAI, Inc.), continues to grow. Unlike Google, AI-generated responses often lack proper sourcing and reliability [75]. Given the evolving role of AI in knowledge acquisition, structured training programs are crucial to helping medical students and physicians navigate these resources effectively. Additionally, integrating verified sources—such as Google Scholar or PubMed—directly into general search engine results for medical queries could further support evidence-based learning and decision-making. General search engines could further support specialized medical queries by suggesting relevant medical keywords based on the task at hand. This approach would help medical students and physicians—who are already familiar with general search engine interfaces—find the required information more quickly than on PubMed, which they often find difficult to navigate efficiently. Physicians’ preference for traditional search engines over PubMed may stem from their perception that PubMed is not well-suited to the practical demands of clinical medicine. General practitioners, in particular, require rapid access to high-quality content and do not have the time to sift through an overwhelming volume of information or conduct an extensive evaluation process [11]. Ideally, search engines should enhance usability by providing clear and precise indicators about the credibility and nature of retrieved results directly on the search results page. This includes specifying whether the content has undergone peer review and clarifying the level of empirical evidence presented in the source. Future research in design ergonomics and medical informatics should focus on developing and testing the impact of such enhanced search interfaces on physicians’ search performance.

### Limitations

This study had some limitations. The small sample size suggests that replicating the study with a larger and more diverse group of participants—including medical students, residents, and both junior and senior physicians—would be valuable. Given that physicians must continuously update their knowledge throughout their careers, future research should explore how search behaviors (eg, keyword formulation, websites visited) evolve at different professional stages, particularly in the context of emerging AI tools.

In addition to better distinguishing the impact of domain knowledge versus information search skills, future research could compare the performance of students in library and information science with that of medical students and residents. As library and information science students have specialized expertise in information retrieval and have outperformed computer science students in prior studies [33], such a comparison could provide further insights into the relationship between search expertise and domain-specific knowledge.

Although this study was conducted remotely—potentially raising concerns about data quality due to limited experimental control—the findings align with previous research. Notably, the search times recorded for medical students and residents in this study were consistent with those reported in prior studies [2]. Moreover, the analysis of search times did not reveal any significant outliers, further supporting the reliability of the collected data.

### Conclusions

The findings of this experimental study have significant implications for both current and future physicians.

A surprising finding was the limited use of evidence-based resources by medical students and residents. This may be due to the challenge of extracting relevant information from a high volume of irrelevant results, as previously documented [64]. This finding highlights the need for enhanced training programs, both at the university and at the postgraduate levels, to equip health care professionals with the skills necessary to effectively utilize evidence-based resources. These programs should focus on improving information search skills, including the efficient use of evidence-based databases and the critical evaluation of online health information. This could include instruction on HONcode principles, which help distinguish between reliable and unreliable sources based on factors such as authorship, funding sources, and URL domains. Beyond their clinical practice, knowledge of source evaluation criteria could also assist physicians in guiding patients who seek medical information online. This is especially important with the rise of AI tools, which are fundamentally reshaping how information is accessed and interpreted.

Training physicians and future physicians to effectively use these tools will also encourage them to expand the range of websites they consult. Rather than limiting themselves to familiar sources, such as hospital websites, they will be better equipped to explore a broader array of credible medical resources.

## Appendix

Multimedia Appendix 1 Examples of answers provided by students-residents in medicine and students in computer science with their description.

## Abbreviations

AI: artificial intelligence
DV: dependent variable
FAIR: Findable, Accessible, Interoperable, and Reusable
HON: Health On the Net
IV: independent variable
MD: Doctor of Medicine
NHS: National Health Service
PhD: Doctor of Philosophy
RQ: research question
