# Supplementary material for: The Influence of Medical Expertise and Information Search Skills on Medical Information Searching: Comparative Analysis From a Free Data Set
Source: JMIR Form Res. 2025 Apr 17;9:e62754. doi: 10.2196/62754 (PMC12046262; doi:10.2196/62754)
Supplement: Multimedia Appendix 1 [file formative_v9i1e62754_app1.docx]

Table 2. Dependent variables for RQ1 with regard to the search task and expertise domain (means and standard deviations).

* indicates significant differences between students-residents in medicine and students in computer science

|  | Group of students | Learning task | Diagnostic task | Treatment task |
| --- | --- | --- | --- | --- |
|  |  | Mean (SD) | Mean (SD) | Mean (SD) |
| **DV1. Search time**  **(sec.)** | **Medicine** | 548 (474) | 705 (444) | 634 (407) |
|  | **Computer Science** | 520 (373) | 1099 (1085) | 936 (699) |
| **DV2. New keywords related to medicine (mean number)** | **Medicine** | 1.80 (0.91)* | 4 (4.19) | 4.8 (4.13) |
|  | **Computer Science** | 0.3 (0.48)* | 2 (2.35) | 2.7 (3.97) |
| **DV3. Correct answers or relevant information provided (mean number)** | **Medicine** | 3.9 (.31)* | 0.9 (0.31)* | 0.85 (0.15)* |
|  | **Computer Science** | 3.5 (.52)* | 0 (0)* | 0.1 (0.31)* |
